# A Remote Monitoring System to Optimize the Home Management of Oral Anticancer Therapies (ONCO-TreC): Prospective Training–Validation Trial

**DOI:** 10.2196/27349

**Published:** 2022-01-26

**Authors:** Alessandro Passardi, Flavia Foca, Orazio Caffo, Carlo Alberto Tondini, Alberto Zambelli, Roberto Vespignani, Giulia Bartolini, Francesco Giulio Sullo, Daniele Andreis, Marco Dianti, Claudio Eccher, Enrico Maria Piras, Stefano Forti

**Affiliations:** 1 Department of Medical Oncology IRCCS Istituto Romagnolo per lo Studio dei Tumori “Dino Amadori” Meldola Italy; 2 Unit of Biostatistics and Clinical Trials IRCCS Istituto Romagnolo per lo Studio dei Tumori “Dino Amadori” Meldola Italy; 3 Department of Medical Oncology Azienda Provinciale per i Servizi Sanitari Trento Italy; 4 Department of Medical Oncology Azienda Socio-Sanitaria Territoriale “Papa Giovanni XXIII” Bergamo Italy; 5 IT Service IRCCS Istituto Romagnolo per lo Studio dei Tumori “Dino Amadori” Meldola Italy; 6 Center for Information and Communication Technology, eHealth Unit Fondazione “Bruno Kessler” Trento Italy

**Keywords:** adherence, oral anticancer drug, mHealth, ONCO-TreC, electronic diary

## Abstract

**Background:**

A platform designed to support the home management of oral anticancer treatments and provide a secure web-based patient–health care professional communication modality, ONCO-TreC, was tested in 3 cancer centers in Italy.

**Objective:**

The overall aims of the trial are to customize the platform; assess the system’s ability to facilitate the shared management of oral anticancer therapies by patients and health professionals; and evaluate system usability and acceptability by patients, caregivers, and health care professionals.

**Methods:**

Patients aged ≥18 years who were candidates for oral anticancer treatment as monotherapy with an Eastern Cooperative Oncology Group performance status score of 0 to 1 and a sufficient level of familiarity with mobile devices were eligible. ONCO-TreC consisted of a mobile app for patients and a web-based dashboard for health care professionals. Adherence to treatment (pill count) and toxicities reported by patients through the app were compared with those reported by physicians in medical records. Usability and acceptability were evaluated using questionnaires.

**Results:**

A total of 40 patients were enrolled, 38 (95%) of whom were evaluable for adherence to treatment. The ability of the system to measure adherence to treatment was high, with a concordance of 97.3% (95% CI 86.1%-99.9%) between the investigator and system pill count. Only 60% (3/5) of grade 3, 54% (13/24) of grade 2, and 19% (7/36) of grade 1 adverse events reported by physicians in the case report forms were also reported in the app directly by patients. In total, 94% (33/35) of patients had ≥1 app launch each week, and the median number of daily accesses per patient was 2. Approximately 71% (27/38) and 68% (26/38) of patients used the app for messages and vital sign entering, respectively, at least once during the study period.

**Conclusions:**

ONCO-TreC is an important tool for measuring and monitoring adherence to oral anticancer drugs. System usability and acceptability were very high, whereas its reliability in registering toxicity could be improved.

**Trial Registration:**

ClinicalTrials.gov NCT02921724; https://www.clinicaltrials.gov/ct2/show/NCT02921724

## Introduction

During the last few decades, oral anticancer drugs, either alone or in combination with intravenous treatments, have occupied an increasingly important space in oncohematology. In addition to traditional hormonal and cytotoxic drugs, new formulations targeting specific genetic mutations (usually referred to as tyrosine kinase inhibitors [TKIs]) have been widely developed [1,2]. The advantages of oral therapies include improved outcomes in several tumor types and a reduction in the workload needed for nurses in terms of administration and intravenous infusion. Moreover, patients generally prefer this type of administration as it enables them to maintain their normal lifestyle [3]. However, alongside these indisputable benefits, some critical issues regarding the use of oral treatments have emerged, especially in relation to treatment adherence and patient safety [4]. For example, toxicities may not be reported, major drug interactions may be overlooked, and self-administration may expose patients to the risk of over- or undertreatment.

Several studies addressing adherence to oral treatments have reported variable rates, some as low as 6% [5-9]. Moreover, adherence levels appear to influence specific clinical and health care outcomes, such as cancer progression, inpatient days, health care resource use and costs, and even survival [10-15]. Numerous variables have been correlated with nonadherence in relation to patients (eg, age and beliefs about medication), treatments (eg, toxicity and complex schedules), health care professionals (eg*,* empathy and communication skills), and the health care system (eg, communication problems with cancer centers) [16-18]. Consequently, methods that can be used to increase adherence levels include patient education and improved communication between health care professionals and patients [19,20].

Patient-centered approaches and mobile health care solutions such as web-based and mobile apps have proven to be useful tools for optimizing the home management of cancer patients. In this context, by involving patients and health care professionals in participatory design techniques (ie, focus group sessions and joint reviews), we were able to customize and adopt an existing health care system (already used for the remote monitoring of patients with asthma, type 1 diabetes, and hypertension) that met the needs of home management and remote monitoring of oral anticancer therapy [21]. We have conducted a prospective training–validation, interventional, nonpharmacological, multicenter study on a new platform called ONCO-TreC for oral anticancer therapy. The overall aims of the trial are to customize the platform; assess the ability of the system to support patients and health care professionals in the shared management of oral anticancer therapies (thus improving adherence and the home management of side effects); and evaluate the usability and acceptability of the system by patients, caregivers, and health care professionals.

## Methods

### ONCO-TreC

ONCO-TreC consists of a mobile app for patients and a web-based dashboard for health care professionals. Clinicians enter the details of medication schemes through the dashboard, set reminders, monitor for adherence to treatment and adverse events (AEs), and communicate with patients via a secure messaging system. The app provides patients with a visual reminder of cancer therapy, a reminder of concomitant drugs to be taken, an easy-to-use AE reporting system, a diary of vital signs, and a messaging system. A detailed description of ONCO-TreC and the trial design has been previously reported [22]. All the cancer centers involved in the study used electronic health records, and although the ONCO-TreC system was designed to be fully integrated into the health records, it was still not connected at this experimental stage. Thus, the investigators consulted the medical records and dashboard separately.

### Patients

A total of 80 patients were considered for the ONCO-TreC trial, comprising 25% (20/80) of patients in the training step and 75% (60/80) patients in the validation step. Eligible patients were required to meet the following criteria: (1) adults aged ≥18 and <75 years of either sex, (2) Eastern Cooperative Oncology Group performance status score of ≤1, (3) candidates for oral anticancer treatment with capecitabine or sunitinib as monotherapy (adjuvant and advanced settings allowed), (4) ability to manage the mobile app after a basic training course held at baseline, (5) clear understanding of the Italian language, and (6) written informed consent. Patients who were also receiving intravenous anticancer treatment or experimental drugs were excluded.

At the time of treatment prescription, the health care professionals provided information on treatment-related side effects and the use and functions of the app. The patients were advised to manually insert data into the system at least once a day. They were seen at study centers every 6 weeks, when clinicians compared adherence and toxicity data entered into the system with those directly reported during the hospital visit and with drug accountability. If the patients were having technical difficulties with the mobile app, the training was repeated. The patients remained under observation until treatment interruption (because of disease progression, unacceptable toxicity, death, or discontinuation) or for ≤6 months.

At the end of the training step (first 20 patients), the results were shared among the centers, the system was fine-tuned, and the study protocol was amended. The sample size of the validation step was reduced from 60 to 20 patients, which is considered a sufficient number to validate the system in a clinical setting. The inclusion criteria were expanded, allowing for the inclusion of patients aged >75 years and those being treated with other TKIs (regorafenib, sorafenib, pazopanib, everolimus, erlotinib, gefitinib, afatinib, axitinib, or crizotinib). The decision to reduce the sample size of the validation step from 60 to 20 patients was made because the analysis of the training step had already highlighted excellent usability and high patient satisfaction. Conversely, to have more efficacy data, it was deemed necessary to design a larger randomized study comparing the effectiveness of the system with that of standard clinical practice.

### Outcome Measures

To assess the system’s ability to monitor adherence, the number of pills counted by the system (entered by the patients at home) was compared with that of residual pills returned by the patients during the hospital visit and counted by the physicians. Patients who took ≥90% of the total drug dose as per the study protocol were defined as adherent. The proportions of adherent patients according to the app and to the pill count were compared. AEs were reported and graded on a daily basis by patients through the app, whereas during study visits, the oncologist recorded the highest grade of each AE per cycle in medical records. The reliability of the system for registering toxicity was evaluated by comparing the type and grade of toxicity indicated by the system with those registered during the clinical visits. The quality of the system was considered adequate if all grade 3 toxicities and ≥80% of grade 2 toxicities reported by the patient at the time of the visit were recorded in the app. In total, 2 validated questionnaires, 1 on perceived levels of quality of life (Functional Assessment of Cancer Therapy-General [FACT-G]) and the other on anxiety (Hospital Anxiety and Depression Scale [HADS]), were administered at baseline and at the end of the study to assess health-related quality of life and anxiety and depression levels in the enrolled patients [23,24]. Given that a review of the literature did not identify any existing validated questionnaires for assessing system usability and acceptability by patients, 2 ad hoc questionnaires were developed by the team of investigators to analyze patient expectations with regard to the system (administered at baseline) and to evaluate system acceptability and communication between patients and cancer centers (administered at week 6 and at the end of treatment). The first questionnaire aimed to establish a baseline with regard to patient communication with the oncology department in terms of the means used (eg, frequency of emails or phone calls) and the adequacy of responses to questions asked. The second questionnaire, distributed at the end of the study, aimed to assess the changes brought about by the use of ONCO-TreC. The overall usability of the system was assessed using the System Usability Scale (SUS) questionnaire, a validated 10-item Likert scale that provides a rapid, reliable subjective evaluation [25]. The SUS was administered at the end of the study to ensure that the patients had had sufficient time to familiarize themselves with the digital device. All the questionnaires were paper-based and self-completed by patients.

### Statistical Analysis

A formal sample size calculation for the prospective study was not performed owing to the lack of preliminary data and the exploratory intent of the study. Frequencies were calculated for categorical variables. For continuous variables, median (minimum to maximum or IQR) or mean and SD were shown. The Wilcoxon and Fisher tests were used to evaluate the difference between the ratios of days of use to total days in different groups of patients. The following tests were used for the comparison between the baseline and end-of-study questionnaires: Wilcoxon matched-pairs signed-ranks for the FACT-G and Stuart-Maxwell for the HADS.

## Results

### System Fine-tuning

The study was activated on May 29, 2015. The TreC system, originally designed for remote monitoring of patients with asthma, type 1 diabetes, and hypertension, was customized to meet the home management and remote monitoring needs of patients with cancer treated with the cytotoxic drug capecitabine or the TKI sunitinib. From June 2015 to December 2015, the system, in particular the mobile app for patients, was fine-tuned through participatory research to comply with the clinical practice regulations of the participating centers. Face-to-face participatory design sessions were conducted with study researchers and patient representatives, the results of which were used to modify the system. Of note, a shared revision of the Common Terminology Criteria for Adverse Events version 4.03, based on health literacy and patient-reported outcome principles, was included in the system to support patient self-reporting of AEs (Multimedia Appendix 1). Some customized suggestions for the management of side effects, differentiated according to the degree of toxicity, were also implemented.

### Patients

From January 2016 to July 2018, a total of 40 patients (20/40, 50% in the training step and 20/40, 50% in the validation step) were enrolled in the ONCO-TreC study from 3 cancer centers in different Italian regions. Patient characteristics are reported in Table 1. The median age was 66 years (range 42-82 years), and 8 patients were aged >75 years. Most were women (24/40, 60%) and had an Eastern Cooperative Oncology Group performance status score of 0 (31/40, 78%) and ≥1 comorbidity (32/40, 80%). As expected, cardiovascular and metabolic or endocrine comorbidities were the most frequent (22/40, 55% and 16/40, 40%, respectively). Oral anticancer drugs comprised mainly capecitabine (23/40, 58%), regorafenib (7/40, 18%), and sunitinib (6/40, 15%). Approximately 68% (27/40) of patients were being treated for advanced disease, and 44% (16/40) had been heavily pretreated. The patients used the app for a mean of 4.4 (SD 8.0) months.

**Table 1:** Patient characteristics (N=40).

Variable		Values
Age at study registration (years), median (range)		66 (42-82)
**Gender, n (%)**		
	Male	16 (40)
	Female	24 (60)
**ECOG^a^ (performance status), n (%)**		
	0	31 (78)
	1	9 (23)
**Comorbidity, n (%)**		
	Yes	32 (80)
	No	8 (20)
**Type of comorbidity, n (%)**		
	Cardiovascular	22 (55)
	Pulmonary	4 (10)
	Gastrointestinal and hepatobiliary	7 (18)
	Metabolic and endocrine	16 (40)
	Musculoskeletal	2 (5)
	Renal or urinary tract	3 (8)
	Allergy	4 (10)
	Neurological and psychiatric	5 (13)
	Other comorbidities	4 (10)
**Site of disease, n (%)**		
	Colorectum	15 (38)
	Pancreas	5 (13)
	Lung	3 (8)
	Breast	4 (10)
	Biliary tract	2 (5)
	Kidney	5 (13)
	Liver	1 (3)
	Unknown	5 (13)
**Oral anticancer drug, n (%)**		
	Capecitabine	23 (58)
	Regorafenib	7 (18)
	Sunitinib	6 (15)
	Afatinib	2 (5)
	Sorafenib	1 (3)
	Gefitinib	1 (3)
**Setting, n (%)**		
	Adjuvant	10 (25)
	Advanced	30 (75)
**Previous therapy, n (%)**		
	None	14 (35)
	1	6 (15)
	≥2	16 (40)
	Unknown	4 (10)

^a^ECOG: Eastern Cooperative Oncology Group.

### Treatment Adherence

Adherence to treatment, calculated according to pill count and registered in a case report form (CRF), was available for 38 patients. Of those 38 patients, 32 (84%) patients were defined as adherent to treatment, 1 (3%) patient had a residual drug that was justified (treatment discontinuation advised by the oncologist because of toxicity), and 5 (13%) patients were defined as nonadherent to treatment. Adherence to treatment according to ONCO-TreC was as follows: 87% (33/38) of patients were adherent, 3% (1/38) were justified nonadherent, and 11% (4/38) were nonadherent. A patient in the nonadherent group reported regular drug intake in the app even during the days of discontinuation. A concordance of 97.3% (95% CI 86.1%-99.9%) was observed between the CRF and app-reported adherence.

### Toxicity Monitoring

Of the 40 enrolled patients, 35 (88%) were evaluable for toxicity. A total of 718 AEs were registered on ONCO-TreC by 18 patients, and 98 AEs were registered for 29 patients by physicians. In total, 1 patient reported AEs in the system but not during the hospital visits. Conversely, of the 29 patients reporting AEs during the study visits, 12 (41%) did not enter any AE information into the system. Table 2 summarizes the AEs reported by patients during study visits and registered in CRFs and those entered by the patients themselves into the app based on grade of severity. Of the 5 grade 3 AEs, 24 grade 2 AEs, and 36 grade 1 AEs reported by physicians in CRFs, 3 (60%), 13 (54%), and 7 (19%) cases, respectively, were also reported in the system by patients.

**Table 2:** Summary of adverse events reported in case report forms (CRF) or the app by grade of severity (N=35).

Adverse event	Registered events in the app, n (%)				Registered events in CRFs, n (%)		
	Grade 1 (n=45)	Grade 2 (n=26)	Grade 3 (n=4)	Grade 1 (n=36)		Grade 2 (n=24)	Grade 3 (n=5)
Asthenia or fatigue	5 (14)	5 (14)	1 (3)	3 (8)		7 (19)	1 (3)
Nausea or vomiting	14 (40)	5 (14)	0 (0)	4 (11)		8 (23)	1 (3)
Rash	1 (3)	0 (0)	0 (0)	1 (3)		0 (0)	0 (0)
Paronychia	0 (0)	0 (0)	0 (0)	1 (3)		0 (0)	0 (0)
Anorexia	1 (3)	1 (3)	1 (3)	4 (11)		1 (3)	0 (0)
Mucositis	3 (8)	3 (8)	1 (3)	1 (3)		3 (8)	1 (3)
Skin toxicity	3 (8)	2 (6)	0 (0)	3 (8)		1 (3)	0 (0)
Nervous system disorders	1 (3)	0 (0)	0 (0)	3 (8)		0 (0)	0 (0)
Alopecia	2 (6)	2 (6)	0 (0)	1 (3)		0 (0)	0 (0)
Hand and foot syndrome	3 (8)	3 (8)	1 (3)	4 (11)		2 (6)	2 (6)
Conjunctivitis	2 (6)	0 (0)	0 (0)	1 (3)		0 (0)	0 (0)
Diarrhea	8 (23)	4 (11)	0 (0)	5 (14)		2 (3)	0 (0)
Fever	0 (0)	0 (0)	0 (0)	3 (8)		0 (0)	0 (0)
Pruritus	2 (6)	1 (3)	0 (0)	2 (6)		0 (0)	0 (0)

### System Use

A total of 5186 accesses to the app were made by 35 patients. Of these35 patients, 33 (94%) patients launched the app at least once a week, whereas only 2 (6%) patients showed a lower frequency (twice or 5 times in 90 days of observation). The median number of accesses per patient per day was 2 (range 1-30 and IQR 1-3), and approximately 25% (9/35) of the patients accessed the system >3 times a day.

The distribution of app launches according to the time slots is presented in Table 3. Most patients used the app in the morning (2428/5186, 46.81% of launches from 6 to 11:59 AM), an expected result as the mobile app reminded patients to take their medications and enter vital signs mainly at this time. There were no significant differences in the rate of app use related to sex, age, or anticancer treatment (Table 4).

At each trial site, the health care professionals (medical oncologist or nurse) involved in the study accessed the system every 24 to 48 hours and before each study visit to check patient status (data not shown).

**Table 3:** Time slots of app launches, conversation starts, and vital sign entering.

Time	App launches (n=5186), n (%)	Conversations (n=100), n (%)	Vital signs (n=1757), n (%)
From 6 to 11:59 AM	2428 (46.98)	54 (54)	1085 (61.75)
From noon to 5:59 PM	1143 (22.11)	31 (31)	173 (9.85)
From 6 to 11:59 PM	1524 (29.49)	13 (13)	143 (8.14)
From midnight to 05:59 AM	91 (1.76)	2 (2)	356 (20.26)

**Table 4:** System usability (app launches, vital sign entering, and messages) according to patient characteristics.

Characteristic		App launches					Vital sign entering							Messages				
		Patients, n (%)	Median days of use/total days, % (IQR)	*P* value (Wilcoxon test)		Patients, n (%)		Median days of use/total days, % (IQR)		*P* value (Wilcoxon test)		None, n (%)			≥1, n (%)		*P* value (Fisher exact test)	
Overall patients		35	67 (39-85)	N/A^a^		26		61 (8-100)		N/A		11 (29)			27 (71)		N/A	
**Gender**					.61						.29							.30
	Female	19	76 (39-89)			14		54 (9-100)				8 (73)			14 (52)			
	Male	16	67 (32-77)			12		61 (6-100)				3 (27)			13 (48)			
**Age (years)**					.44						.29							.07
	<66	16	56 (28-83)			13		95 (9-100)				8 (73)			11 (41)			
	≥66	19	74 (40-85)			13		27 (6-90)				3 (27)			16 (59)			
**Drug**					.33						.32							.29
	Capecitabine	18	72 (40-89)			12		10 (4-100)				8 (73)			13 (48)			
	TKIs^b^	17	67 (19-80)			14		87 (27-100)				3 (27)			14 (52)			

^a^N/A: not applicable.^b^TKI: tyrosine kinase inhibitor.

### Vital Sign Entering

Approximately 68% (26/38) of patients entered ≥1 parameter during the study, the main items being blood pressure (22/26, 85%), pulse rate (9/26, 35%), weight (18/26, 69%), and body temperature (8/26, 31%). Most parameters were entered in the morning (1085/1757, 61.75% from 6 to 11:59 AM), and some were registered in the afternoon (173/1757, 9.84% from noon to 5:59 PM), in the evening (143/1757, 8.14% from 6 to 11:59 PM), and during the night (356/1757, 20.26% from midnight to 5:59 AM), the latter mainly because of patients entering the information in the early hours of the morning (Table 3).

The rate of app use for vital sign entering, calculated as the number of days in which ≥1 parameter was registered with respect to the total number of days of observation, was 61%. There were no significant differences in the rate of vital sign entering related to sex, age, or anticancer treatment. Vital signs were entered more frequently by younger patients (95% <66 years vs 27% ≥66 years) and by patients receiving TKIs (87% vs 10% for capecitabine; Table 4).

### Use of System for Messages

Approximately 71% (27/38) of patients used the app for messages at least once during the study, and 212 messages were generated in 100 conversations between patients and health care professionals. The conversations mainly regarded problems with side effects (59/100, 59%), difficulties with the app (30/100, 30%), and clarification about appointments (11/100, 11%). Most messages were sent during the morning hours (54/100, 54% from 6 to 11:59 AM) or in the afternoon (31/100, 31% from noon to 5:59 PM; Table 3). There were no significant differences in the use of the messaging system related to sex, age, or anticancer treatment. However, elderly patients used the messaging system more frequently (Table 4); 59% (16/27) of the patients who sent ≥1 message were aged >65 years, whereas approximately 73% (8/11) of the patients who did not use the messaging system were aged <65 years.

### Alarm System

The ONCO-TreC was endowed with a rule-based alarm system [21,22] defined by our oncologists and drawing on state-of-the-art knowledge in the area of cancer. Alarms went off at least once for 23 patients, and a total of 150 alarms were activated. Among the reasons for alarm activation were data not entered for 3 days in a row (22/150, 14.7%), anticancer drugs not taken for 3 days in a row (47/150, 31.3%), systolic blood pressure ≥160 mmHg or diastolic blood pressure ≥100 mmHg (69/150, 46%), and grade ≥3 AEs (12/150, 8%). The median time to resolution by health care professionals was 2 days.

### FACT-G and HADS Questionnaires

A total of 36 patients were evaluated by administering questionnaires at baseline, of whom 34 (94%) completed the FACT-G questionnaire and 35 (97%) completed the HADS questionnaire. Patients showed a mean total score of 69.1 (SD 14.9), reporting better physical well-being (mean 22.5, SD 5.1) but poorer functional well-being (mean 11.4, SD 5.4). Of the 35 cases evaluated by the HADS questionnaire at baseline, 20 (57%) and 19 (54%) were considered to have normal levels of anxiety and depression, respectively.

Data from the FACT-G and HADS questionnaires administered at baseline and at the end of the study were available for only 18 and 20 patients, respectively. In the subgroup of patients who answered both the baseline and posttreatment FACT-G questionnaire, a slight reduction in physical well-being emerged (*P*=.046), whereas the scores for the other subscales did not change substantially. Of the 20 patients with both pre- and posttreatment HADS, 3 (15%) showed an improved anxiety score after treatment, whereas another 3 (15%) had a worse score (*P*=.55). Similar results were reported in the depression subscale, with 4 (20%) patients achieving a better status after treatment, and another 4 (20%) showing a worse status (*P*=.77).

### Usability and Acceptability

The patients did not encounter any significant issues in interacting with the app. The SUS scores indicated an excellent subjective assessment of the usability of the system. The overall score was <68 (the average SUS score) in only a small percentage of patients (11.8%); however, it still denoted an acceptable overall usability of the system. An overall score of >80.3 was recorded for 76.4% of patients, which is considered the threshold of excellence [26]. Of note, few patients reported being *worried* about their ability to use the system before they started the trial, and some mentioned having requested the help of a relative the first time they used it. However, all patients reported being able to use ONCO-TreC by themselves within a short period.

The acceptability of ONCO-TreC and its perceived benefits was explored using 2 ad hoc questionnaires distributed to patients at the end of the trial. The overall satisfaction was *very satisfied* (10/20, 50%), *moderately satisfied* (9/20, 45%), *slightly satisfied* (5/20, 5%), and *not satisfied* (0/20, 0%). The patients reported an overall positive effect on several aspects of health care management, measured using a Likert scale. ONCO-TreC proved useful for self-management purposes and was considered a valuable reminder of when to take therapy (19/20, 95% *strongly agreed* and 1/20, 5% *agreed*) and a useful AE tracker (14/20, 70% *strongly agreed* and 5/20, 25% *agreed*). The questionnaire also highlighted a significantly positive effect on the patient–provider relationship as patients felt that ONCO-TreC reinforced the perception of being continuously monitored by providers (18/20,90% *strongly agreed* and 2/20,10% *agreed*). An overall positive evaluation was also recorded for the messaging system as a useful communication tool (12/20, 60% *strongly agreed* and 4/20, 20% *agreed*).

### Patient–Provider Communication

ONCO-TreC modified the way in which patients were able to communicate with health care professionals. An ad hoc questionnaire was developed to investigate the communication channels used by patients before and after the introduction of the system. Of the 35 patients who completed the baseline questionnaire, only 20 (57%) returned to the questionnaire at the end of the treatment. Table 5 shows the results of the pre- and posttreatment evaluations for this subgroup. Although the small number of participants and the brevity of the study period did not allow for any definitive conclusions to be drawn about this, our results suggest that the ONCO-TreC messaging system helped reduce all forms of direct contact that would have resulted in a disruption of the workflow in a health care setting (eg, phone calls to a switchboard or physician and physically going to the hospital). Although only 71% (27/38) of the patients used the messaging system integrated into the platform, our findings indicate that it minimized synchronous interactions by favoring asynchronous communication.

**Table 5:** Channels to contact providers before and after the introduction of ONCO-TreC (N=20).

Contact with the oncology department in the previous couple of months	Often or sometimes, n (%)		Rarely or never, n (%)	
	Before	After	Before	After
Phone call to switchboard	10 (50)	7 (35)	10 (50)	13 (65)
Phone call to physician	6 (30)	4 (20)	14 (70)	16 (80)
Email to physician	4 (20)	2 (10)	16 (80)	18 (90)
Going to the department	13 (65)	7 (35)	7 (35)	13 (65)
ONCO-TreC system	N/A^a^	7 (35)	N/A	13 (65)
Other	1 (5)	0 (0)	19 (95)	20 (100)

^a^N/A: not applicable.

## Discussion

### Principal Findings

This project aimed to fine-tune and validate an oral anticancer therapy monitoring system in terms of its ability to increase adherence to treatment, improve home management of treatment side effects, and enhance patient or health care professional communication. Adherence is normally considered as the percentage of the prescribed treatment dose actually taken by the patient over a specified period. The most common and simple method used to measure adherence other than direct patient questioning is pill count (ie, counting the number of pills that remain in a patient’s medication bottles or vials). Although the simplicity and empirical nature of this method are attractive to many investigators, the method is subject to problems as patients may switch medicines between bottles and discard pills before visits to appear to be following the regimen. Improving communication between health care professionals and patients through new technologies such as reminders and PDAs might thus be an effective strategy to increase adherence. In this trial, adherence was measured both by pill count and by the number of pills counted by the system (entered every day by the patients at home). Adherence was very high and may have been due, among other factors, to the reminder system integrated into the app, which was considered very useful by virtually all patients. However, the small sample size and absence of a control arm did not allow us to draw definitive conclusions about the efficacy of the system in improving adherence. The ability of the system to measure adherence to treatment was also high, with a concordance of 97.3%.

The reliability of the platform to register toxicity was also investigated by comparing the type and grade of toxicities recorded in the system with those reported by patients during the clinical visits. The quality of the system proved inadequate in that only a fraction of AEs reported by physicians in CRFs were also recorded in the app by patients. This negative result was probably related to some patients not using the app to register toxicity because of technical difficulties, underestimation of the importance of the AE, or other unknown causes. In fact, of the 29 patients reporting AEs during the study visits, only 17 (59%) entered information on the events into the system. Conversely, the patients using the app for AE entering were very meticulous and provided a large amount of data (718 AEs), enabling clinicians to create an accurate, day-to-day reconstruction of the toxicity trend over time. It can thus be hypothesized that, although ONCO-TreC has a high capacity for detecting and monitoring AEs, patients need to be made aware of the importance of this complex part of the system and trained to use it correctly.

The system helped reshape the patient–provider interaction between clinical visits, enabling some synchronous communication to be turned into asynchronous contact through the messaging system. Even though the reshaping of communication was not a primary end point of this study, the decrease in phone calls and patients physically going to the hospital in favor of texting through the platform could make communication more manageable during home oral anticancer treatment. Phone calls and visits tend to disrupt the workflow of hospital departments, whereas asynchronous communication allows providers to make good use of downtime or even define a specific time slot to interact with patients at home. Partial confirmation of the potential usefulness of messaging can be obtained by comparing it with alarm management. In fact, alarms are the counterpart of messages. The former go off automatically when the rule-based system detects a potential harmful pattern, whereas the latter are sent by patients because they need to communicate something. However, in this experimental phase, both alarms and messages addressed noncritical issues.

The use and usability of the system were also analyzed extensively. Approximately 94% (33/35) of patients launched the app at least once a week, and the median number of daily accesses per patient was 2. Most patients used the app for messages and vital sign entering at least once during the study period. The usability of the system was greatly appreciated by patients, and preliminary results of the training step led to the extension of the age limit for enrollment in the trial. This result was probably due to the use of a simplified interface with visualization tools that patients were familiar with and to the work carried out in the preliminary phase of the study with representatives of patient associations. However, as previously mentioned, only some patients used the most advanced functions of the app, that is, messaging and AE reporting. Thus, other studies are warranted to investigate the usability of each function and determine whether their limited use is a result of their design or a lack of interest on the part of the patients.

### Conclusions

The ONCO-TreC system appears to be an important tool for improving the home management of and monitoring adherence to oral anticancer drugs. Although the usability and acceptability of the system were very high, its reliability in registering toxicity needs to be improved. A phase III trial comparing the ONCO-TreC system with a standard oral anticancer treatment diary is currently recruiting patients (ClinicalTrials.gov ID NCT04826458).

## Appendix

Multimedia Appendix 1 App self-reported items.

## Abbreviations

AE: adverse event
CRF: case report form
FACT-G: Functional Assessment of Cancer Therapy-General
HADS: Hospital Anxiety and Depression Scale
SUS: System Usability Scale
TKI: tyrosine kinase inhibitor
