# Supplementary material for: A Remote Monitoring System to Optimize the Home Management of Oral Anticancer Therapies (ONCO-TreC): Prospective Training–Validation Trial
Source: J Med Internet Res. 2022 Jan 26;24(1):e27349. doi: 10.2196/27349 (PMC8829690; doi:10.2196/27349)
Supplement: Multimedia Appendix 1 [file jmir_v24i1e27349_app1.docx]

App self-reported items.

| Adverse event | Grade 1 | Grade 2 | Grade 3 | Grade 4 |
| --- | --- | --- | --- | --- |
| Asthenia | — | — | — | — |
| Bone pain | — | — | — | — |
| Bruising and bleeding | — | — | — | — |
| Conjunctivitis | — | — | — | — |
| Constipation | — | — | — | — |
| Cough | — | — | — | — |
| Decreased appetite | — | — | — | — |
| Diarrhea | — | — | — | — |
| Difficulty breathing | — | — | — | — |
| Dizziness | — | — | — | — |
| Dry skin | — | — | — | — |
| Dyspepsia | — | — | — | — |
| Hand and foot syndrome | — | — | — | — |
| Hair loss | — | — | — | — |
| Headache | — | — | — | — |
| Hoarseness | — | — | — | — |
| Itch | — | — | — | — |
| Mucositis | — | — | — | — |
| Muscular pain | — | — | — | — |
| Nail inflammation | — | — | — | — |
| Nausea | — | — | — | — |
| Pain | — | — | — | — |
| Rash | — | — | — | — |
| Sensitivity disorders | — | — | — | — |
| Vomiting | — | — | — | — |
